# Introducing a food program to increase muscle mass power in a high school baseball club: perception of players and guardians

**DOI:** 10.1186/1550-2783-8-S1-P11

**Published:** 2011-11-07

**Authors:** Tokie Izaki, Kazue Yamamoto

**Affiliations:** 1 Department of Human Development and Education, Tokaigakuen University, Nakahira Tenpakuku 2-901, Nagoyashi Aichiken 468-8514, Japan; 2 Nagoya City University, Graduate School of Natural Sciences. Yamanohata 1, Mizuhocho Mizuhoku Nagoyashi 467-8501, Japan

## Background

In Japan, many baseball clubs have been trying to increase players’ food intake so that players could increase muscle mass power to obtain better performance. Ways to do this have included increasing protein intake and eating between meals. It is also common in Japan to provide players with a food program which encourages them to eat as much food as they can for 5-7 day. The aim of this supervised program is to increase their food consumption. However, one possible risk is that players develop a strong loathing for food. Therefore, this study targeted the perceptions of players and guardians about a food program.

## Methods

A 10 day food program was introduced by coaches at a high school baseball club. For 10 days, only during lunch time (50-60 minutes), players were under an obligation to eat as much food as they could (mainly carbohydrates, in addition to a lunch box (500-600cal). A questionnaire was administered to high school baseball players (n=43) and their guardians (n=43) to explore how they perceived the amount of food, the change of their food intake and weight (e.g., height 172.37cm, weight 66.75kg on average) and what they thought of the program overall.

## Results

Almost 82% of players reported that the amount of food intake was too much. Regarding the change of weight after the food program, 63% of players (increased 1900g on average, according to players’ self-report) and 53% of guardians reported ‘changed successfully’. Regarding the amount of food intake after the program, 62% of players and 55% of guardians reported ‘increased’. Guardians commented that players realized what amount of food they should intake (43%). Some guardians also explained that enjoying food was not something they paid attention to (13%).

## Conclusion

The majority of players were interested in increasing their weight. Guardians found that it was often difficult to find time to provide this kind of opportunity. Therefore, most players and guardians commented about the program positively. However, there were many considerations related to this intervention such as needing to pay more attention to body fat percentage, muscle mass and the contents of food.

